# Percutaneous Autologous Bone Marrow-Derived Mesenchymal Stromal Cell Implantation Is Safe for Reconstruction of Human Lower Limb Long Bone Atrophic Nonunion

**DOI:** 10.22074/cellj.2016.4866

**Published:** 2016-12-21

**Authors:** Mohsen Emadedin, Narges Labibzadeh, Roghayeh Fazeli, Fatemeh Mohseni, Seyedeh Esmat Hosseini, Reza Moghadasali, Soura Mardpour, Vajiheh Azimian, Alireza Goodarzi, Maede Ghorbani Liastani, Ali Mirazimi Bafghi, Mohamadreza Baghaban Eslaminejad, Nasser Aghdami

**Affiliations:** Department of Regenerative Biomedicine, Cell Science Research Center, Royan Institute for Stem Cell Biology and Technology, ACECR, Tehran, Iran

**Keywords:** Nonunion, Mesenchymal Stromal Cells, Autologous, Bone Marrow

## Abstract

**Objective:**

Nonunion is defined as a minimum of a 9-month period of time since an injury with no visibly progressive signs of healing for 3 months. Recent studies show that application of mesenchymal stromal cells (MSCs) in the laboratory setting is effective for bone regeneration. Animal studies have shown that MSCs can be used to treat nonunions. For the first time in an Iranian population, the present study investigated the safety of MSC implantation to treat human lower limb long bone nonunion.

**Materials and Methods:**

It is a prospective clinical trial for evaluating the safety of using autologus bone marrow derived mesenchymal stromal cells for treating nonunion. Orthopedic surgeons evaluated 12 patients with lower limb long bone nonunion for participation in this study. From these, 5 complied with the eligibility criteria and received MSCs. Under fluoroscopic guidance, patients received a one-time implantation of 20-50×106 MSCs into the nonunion site. All patients were followed by anterior-posterior and lateral X-rays from the affected limb, in addition to hematological, biochemical, and serological laboratory tests obtained before and 1, 3, 6, and 12 months after the implantation. Possible adverse effects that included local or systemic, serious or non-serious, and related or unrelated effects were recorded during this time period.

**Results:**

From a safety perspective, all patients tolerated the MSCs implantation during the 12 months of the trial. Three patients had evidence of bony union based on the after implantation Xrays.

**Conclusion:**

The results have suggested that implantation of bone marrow-derived MSCs is a safe treatment for nonunion. A double-blind, controlled clinical trial is required to assess the efficacy of this treatment (Registration Number: NCT01206179).

## Introduction

According to the American Food and Drug Administration (FDA) in 1988, nonunion, which occurs in approximately 5-10% of fractures, (1) is established when at least nine months has passed since an injury and the fracture does not show any visible signs of healing for three months (2). It is estimated that in the US alone, 7.9 million fractures occur annually (3). Nonunion is generally difficult to treat, in particular those that are atrophic. Even with autologous bone grafting, which is the current treatment for a nonunion, approximately 10% of cases will have major complications; about 40% will suffer from minor complications (4). This type of treatment has a variety of complications and presents a therapeutic challenge to surgeons. There exist numerous alternative under investigation treatments for nonunion (5, 6), however none have thus been approved. In the past few decades, there have been large numbers of studies on stromal cell applications and their effects on the regeneration of body tissues. Mesenchymal stromal cells (MSCs) are defined as non-hematopoietic stromal cells present in the human bone marrow, fat tissue, and muscles. MSCs have multilineage differentiation capabilities (7). This capability is an ideal option for the treatment of bone defects such as a nonunion. A number of experimental model studies have reported the safety and efficacy of MSC applications in treating nonunion (8-12). Zhu et al. (13) reported that osteogenically induced bone marrow stromal cells could repair goat femur defects. In a human study, Xue et al. (14) intravenously infused umbilical cord MSCs into a patient who suffered from nonunion of the humerus and radial nerve injury. They observed that at 60 days after the infusion, the bony gap disappeared and nerve conduction velocity increased with shorter latency and higher amplitude. In another study, Fayaz et al. (15) used MSCs to treat a subtrochanteric femoral nonunion with a broken nail. Based on the above mentioned studies, the current research intended to evaluate the safety of implanted MSCs as a treatment of lower limb human long bone nonunion.

## Materials and Methods

### Patients

**Fig.1:**
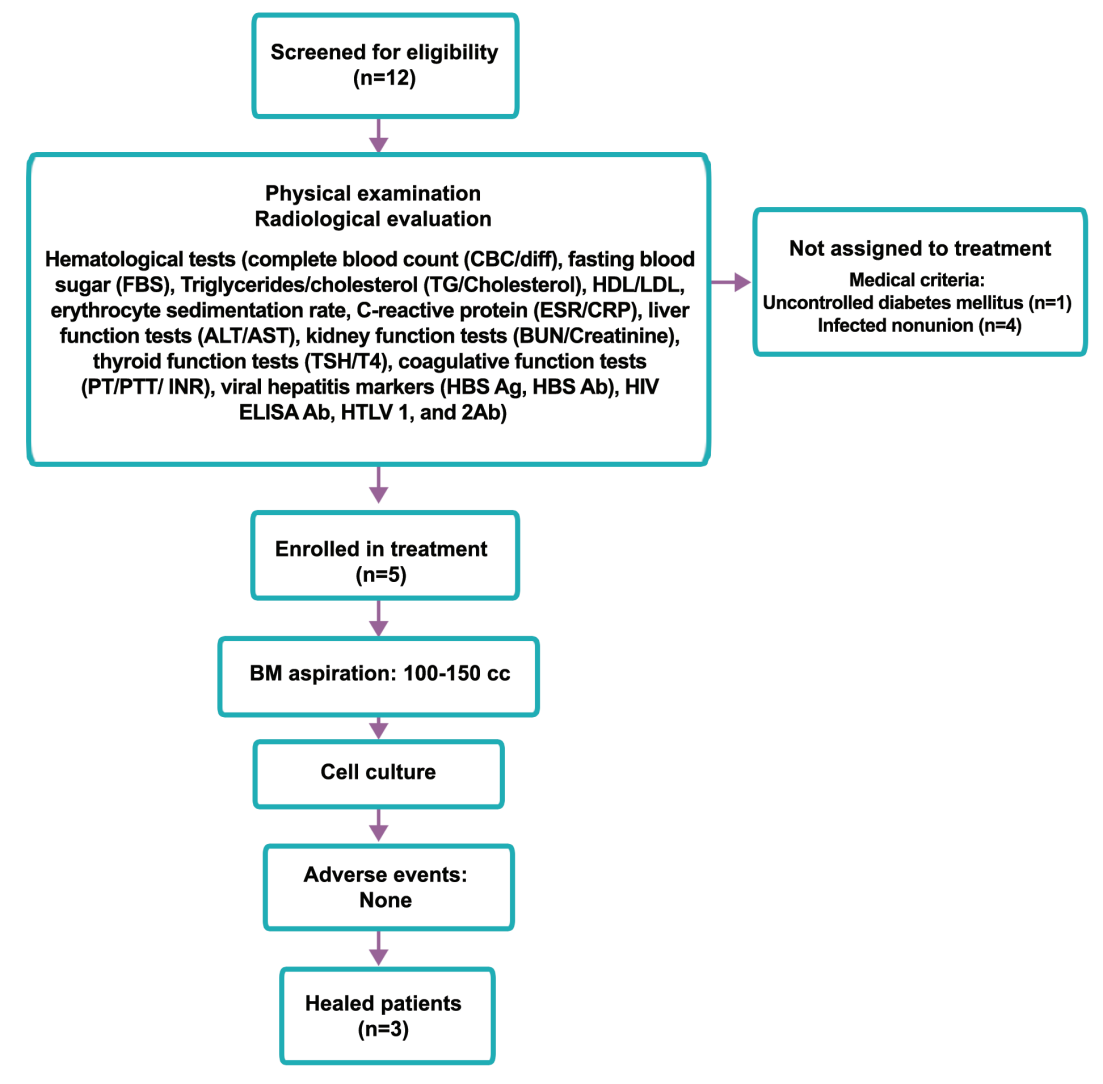
Flowchart of patients. HDL; High density lipoprotein, LDL; Low density lipoprotein, ALT; Alanin aminotransferase, AST; Aspartate aminoternsferase, BUN; Blood urea nitrogen, TSH; Thyroid stimulating hormone, T4; Thyroxine, PT; Prothrombine time, PTT; Partial thromboplastine time, INR; International ration, HBS Ag; Hepatitis B surface Ag, HIV; Human immunodeficiency virus, HTLV; Human T lymphtropic virus, BM; Bone marrow, and MSCs; Mesenchymal stromal cells.

The Ethical Review Board of Royan Institute approved the present prospective clinical trial. Informed consent was taken from all eligible patients before inclusion in the study. Between 2012 and 2013, orthopedic surgeons selected 5 out of 12 patients based on inclusion and exclusion criteria (Table 1, Fig.1).

**Table 1:** Detailed inclusion and exclusion criteria


Inclusion criteria	Exclusion criteria

Age (18 to 65 Y)	Active infection at nonunion
Established nonunion of lower limbs	Inadequate fixation of nonunion
Diaphyseal	Positive viral tests
Atrophic type nonunion	Pregnancy, lactating Chronic, uncontrolled diseases


### Bone marrow aspiration and mesenchymal stromal cell isolation

Each patient underwent surgery on both iliac crests, performed under sterile conditions by a hematologist/ oncologist. Patients received local anesthesia that consisted of a 2% lidocaine solution and sedation by an intravenous infusion of midazolam (Tehran Chemie pharmaceutical Co., Iran, 0.1 mg/kg) and fentanyl (Aburaihan pharmaceutical Co., Iran, 25- 50 mg/100 mm). Mononuclear bone marrow (BM) cells were isolated under sterile conditions according to the density gradient strategy by the Ficoll-Paque open system (Lymphodex, Inno-Train, Ref: 002041600). Next, we isolated and washed the mononuclear cell (MNC) layer in phosphate buffered saline (PBS, Miltenyi Biotech GmbH, Ref: 700-25). Cell count and viability was evaluated with trypan blue staining and confirmed by a NucleoCounter system (ChemoMetec A/S, Denmark). Then, MNCs were cultured under standard culture conditions that consisted of MEM Alpha Medium 1X (Gibco, Cat. No.: 22571) and supplemented with 10% fetal bovine serum (16) Pharma Grade (PAA, Cat. No.: A15-512). The cultured MNCs were subsequently seeded at 1×10^6^MNCs/cm^2^in Millicell HY Flasks (Millicell HY Flask T-600, Cat. No.: PFHYS0616) for a primary culture. Flasks were incubated under predefined conditions of 5% CO_2_ at 37˚C Following the initial 3-4 days, the medium was transferred to new flasks in order to give the floating cells enough time to attach. Non-adherent cells were removed by changing the culture medium after 3-4 days. After 1-2 passages, fibroblast-like cells that had reached 90% confluency were harvested with 0.05% trypsin/EDTA (Gibco, Germany). Cell viability was determined by trypan blue staining as well as the NucleoCounter system prior to the injection.

**Fig.2:**
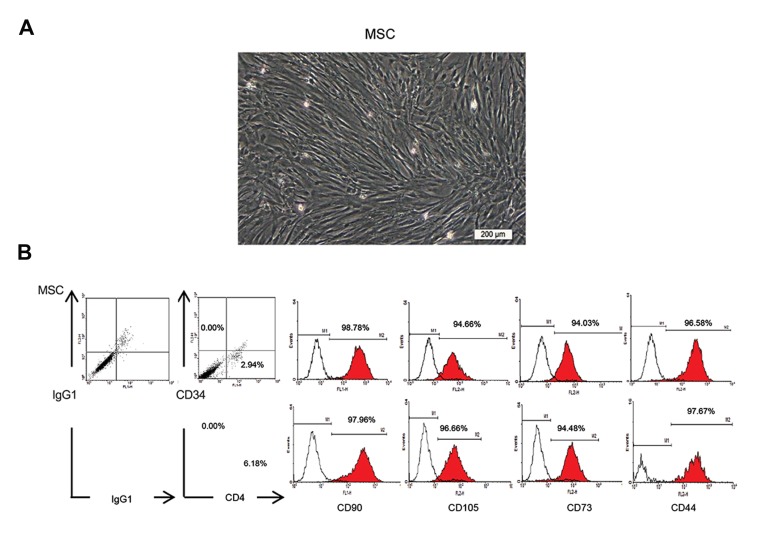
Characterization of passage-1 human bone marrow mesenchymal stromal cells (MSCs). A. Phenotypic appearance of fibroblast-like MSCs after one passage in MSCs injected in patients with nonunion and B. Representative flow cytometric analysis using WinMDI software confirmed the expressions of CD90, CD105, CD73, CD44, and CD45/CD34 surface markers (red lines) on MSCs compared to isotype controls (black lines).

Flow cytometry analysis was performed in order to determine expression of the cell surface markers. The characterization panel consisted of monoclonal antibodies for mesenchymal lineages markers CD90-FITC (Exbio, Cat. No.: 1F-652-T100), CD105-PE Endoglin (BD PharmingenTM, Cat. No.: 560839), CD73-PE (BD PharmingenTM, Cat. No.: 550257), CD44-FITC (BD PharmingenTM, Cat. No.: 555478), and CD45FITC-CD34PE (BD PharmingenTM, Cat. No.: 341071), in addition to the isotype controls MultiMixTM FITC mouse IgG1, PE-mouse IgG1 (Dako, X0932), FITC-mouse IgG2b (Millipore, Cat. No.: MABC006F), and PE conjugated mouse IgG1k (BD PharmingenTM, Cat. No.: 551436). Cells were fixed with 4% paraformaldehyde. Immunophenotyping analysis was performed using the BD FACS Calibur flow cytometry system (BD Biosciences, San Jose, CA, USA). Finally, cells were resuspended in 7 ml normal saline supplemented with 2% human serum albumin (Octalbin, Octapharma, AG, Seidenstrass2 CH-8853 Lachen, Switzerland). Figure 2 shows the characterization of the MSCs. 

### Preparation of cells for implantation

Primary cultures of MSCs were washed with PBS and trypsinized with trypsin/EDTA (0.05%, Gibco, Cat. no.: 25300-062). The cells were suspended in normal saline at a density of 10×10^6^/ml medium and loaded into 10 ml sterile syringes. For each patient, we prepared approximately 20-50×10^6^cells which were subsequently taken to the hospital in a cold (0-4˚C) box in 1-2 ml of normal saline. Each patient received a transcutaneous implantation of the MSC suspension at the nonunion site. An orthopedic surgeon performed the procedure via a fluoroscope.

### Evaluation of adverse events

We categorized all adverse events as local or systemic, serious or non-serious, and related or unrelated. Local adverse events comprised those limited to the nonunion site; systemic ones were considered unrelated to the nonunion site; and serious adverse events included death, neoplasms, infections, pulmonary embolisms, and anaphylactic shock. All patients underwent evaluations for adverse events immediately after the implantation and 3, 6, and 12 months later. At these intervals, X-rays of the affected limb as well as the above mentioned laboratory tests were examined.

## Results

The 5 patients ranged in age from 23 to 55 years. There were 2 females. Demographics and results are shown in Table 2. 

### Clinical and radiological results

**Fig.3:**
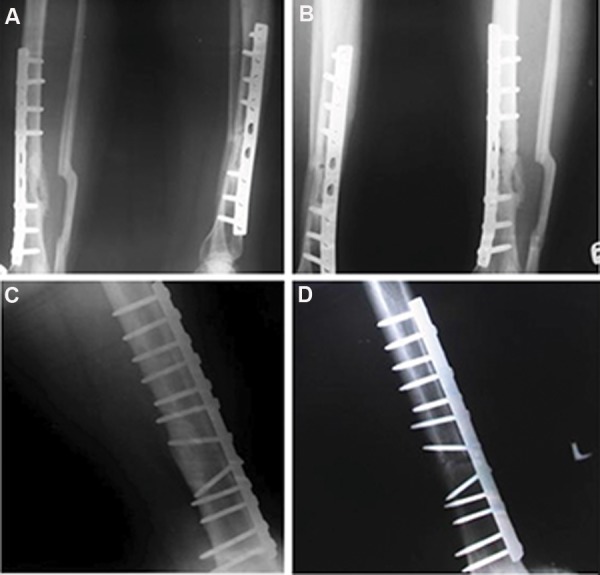
Patients radiographies. A. Anteroposterior and lateral radiographs of a non-united open tibial and fibular fracture of a 32-year-old patient, B. 2 months after using mesenchymal stromal cells (MSCs) with demonstrated radiological signs of healing, C. Lateral radiograph of a non-united femural fracture of a 23-year-old patient before implantation of mesenchymal stromal cells, and D. 6 months after transcutaneous implantation of MSCs with radiological signs of healing.

There were no adverse events as previously mentioned in any of the patients. There was improvement in healing and bone union observed in 3 patients. A 32-year-old woman presented with both bone closed fractures of the leg that had previously been treated unsuccessfully by plating. The nonunion lasted for 12 months. She had a deformity of the lower limb and underwent several corrective surgeries. However, six months after implantation of the MSCs, Xrays showed union at the fracture site (Fig.3A, B). The other patient, a 23-year-old man, had a closed fracture of the femur. He had a nonunion that lasted for two years without any effective treatment. Prior treatments included plating plus internal fixation, which were unsuccessful. One year after implantation of the MSCs, X-rays showed union of the affected bone (Fig.3C, D). The third patient was a 23-year-old man with a closed fracture of the femur after a motor accident with shortening of the lower limb. The fracture was previously treated by plating, which was unsuccessful. The patient had a history of corrective bone grafting which did not lead to healing. However, six months after implantation of the MSCs, union occurred and the bone gap disappeared. The other two patients included a 29-year-old man with a closed fracture of the tibia that had lasted for 6 years and a 55-year-old woman with a closed fracture of the femur and nonunion for 15 months. Neither of these patients benefited from the MSCs implant (Table 2). Of note, we did not observe any adverse effects in any of the patients.

**Table 2:** Demographic and clinical characteristics of the patients included in the study


Case	Age (Y)	Sex	Site	Initial injury	Duration of nonunion	Physical exam	Fracture mobility	Initial treatment	Type of nonunion	Time of nonunion

1	29	M	Tibia	Closed	6 years	Shortening	No	Int. fixation	A	Failed to unite
2	32	F	Tibia and fibula	Closed	12 months	Deformity	No	Plating	A	6 months
3	23	M	Femur	Closed	24 months	Deformity	No	Plating/int. fixation	A	12 months
4	55	F	Femur	Closed	15 months	Shortening	No	Plating	A	Failed to unite
5	23	M	Femur	Closed	7 months	Shortening	No	Plating/ iliacbone graft	A	6 months


## Discussion

Nonunion and its complications are significant concerns for orthopedic surgeons (17). Although surgical techniques and orthopedic procedures have improved, recent literatures note that the failure rate of fracture treatments is higher than previously believed (18, 19). Some of the causes for nonunion are believed to be complex, high- energy traumatic fractures and aggressive treatment of these fractures (20). It is important to find reasons that cause nonunion complications. After a fracture, excess motion, insufficient vascularity, a large bone gap, and infection can potentially cause nonunion (21). Bone regeneration and healing combined with understanding the baseline pathology are necessary for treatment of nonunion patients (22, 23).

Four integral elements for bone repair are osteogenesis, osteoinduction, osteoconduction, and a good osseous environment. These elements are of utmost importance for a bone graft, which is known as the best current treatment and management of nonunion (24). Osteogenesis is the ability of cells to survive transplantation, undergo proliferation and differentiation into viable osteocytes (25). Osteoinduction is the stimulation and activation of the host cells from surrounding tissue to the osteoblasts (26). Osteoconduction is the creation of a stable bone scaffold to support mature osteocytes from the surrounding environment (27). The ideal bone graft incorporates these essential elements into a good osseous environment (28). The current treatment for nonunion which is capable of providing all three elements of bone regeneration is autologous cancellous bone grafting (29). However, bone grafts possess their own complications. Approximately 25% of patients treated with autologous bone grafts have prolonged pain, hematomas, neurologic symptoms, seromas, or infections at the harvest site (4). Another problem for this method is the limitation of the graft amount in quantity and quality, especially in advanced ages (4, 30).

Studies, in order to provide a less morbid yet equally effective alternative, have focused on certain orthobiologics normally found in bone (e.g., MSCs). These stromal cells can be easily and practically applied to the nonunion site. MSCs have inherent properties such as osteoinductivity, by which the surrounding bone cells and tissues participate in the process of repairing the fracture. Attraction and stimulation of osteoprogenitor cells to differentiate into chondrocytes and osteoblasts is a benefit of these stromal cells for bone formation. Unlike the other bioactive products such as bone morphogenic protiens (BMPs), MSCs have osteoconductivity potential that can create a new stable bone scaffold for assisting the bone graft (31).

It is important to evaluate the safety of each novel biological treatment in all populations. Therefore, this study has intended to show the safety of autologous MSC implantation. Results from the present study show that MSCs implantation is a safe technique for treatment of lower limb long bone nonunion. In some cases this treatment can improve the healing process.

The present work was a prospective clinical trial phase 1 study with a small number of cases that aimed to evaluate the safety of MSCs implantation to treat nonunion. Future randomized phase 2 clinical trials would be required to evaluate the efficacy of this treatment.

## Conclusion

Nonunion is a morbid disease of concern to both patients and physicians, and, consequently, affects society. Results from the present study have suggested that the use of MSCs is safe for treating bone nonunion and can be considered an option for orthopedic surgeons to focus on researches in the field of nonunion. The evidence from the present study suggests that MSC implantation is a safe method for treating nonunion. Phase two clinical trials are needed for evaluating its efficacy. The present study has shown that two cases unresponsive to previous treatments and one patient who underwent the iliac bone grafting method were cured by implantation of MSCs. Use of MSCs is a rapidly expanding focus of researches in all fields of medical sciences, in particular, the field of orthopedics and needs further scientific investigations. Future randomized phase 2 clinical trials are absolutely necessary to evaluate the efficacy of MSCs implantation for the treatment of long bone nonunion.
